# Transcriptomic Profiling of Duodenal Epithelium Reveals Temporally Dynamic Impacts of Direct Duodenal Starch-Infusion During Dry Period of Dairy Cattle

**DOI:** 10.3389/fvets.2019.00214

**Published:** 2019-07-02

**Authors:** Cong-Jun Li, Shudai Lin, María Jose Ranilla-García, Ransom L. Baldwin

**Affiliations:** ^1^Animal Genomics and Improvement Laboratory, Agricultural Research Service, Beltsville Agricultural Research Center, USDA, Beltsville, MD, United States; ^2^Department of Animal Genetics, Breeding and Reproduction, College of Animal Science, South China Agricultural University, Guangzhou, China; ^3^Departamento de Producción Animal, Instituto de Ganadería de Montaña, CSIC-Universidad de León, Campus de Vegazana, León, Spain

**Keywords:** ruminant, dairy, duodenal, RNA seq analysis, starch, dry period, biopsy

## Abstract

Previous research has demonstrated a positive relationship between dietary Metabolisable Energy Intake (MEI) and increased maintenance energy costs associated with the visceral tissues. Limitations in understanding this relationship include a lack of access to samples to assess regulatory control of the putative response gastrointestinal tissues to nutrients. This experiment was conducted with a single nutrient (starch hydrolysate) infused (7 d) directly into the intestine to mimic typical changes in post-ruminal starch delivery in dairy production settings. Duodenal epithelial samples collected via biopsy were evaluated using next-generation sequencing technology (RNA-Seq) to validate the use of this approach for the profiling and comparison of the transcriptome of cattle intestinal epithelial tissues. Samples of intestinal epithelial tissue were collected prior to and during the infusion of starch hydrolysate. Biopsies were collected on day 0 before and day 1, day 3, and day 7 during the infusion. Additionally, samples were collected on day 1 and day 7 after infusion was discontinued (Day 8 and Day14 of the experiment). Evaluation of RNA-seq data revealed dynamic changes in global gene expression during infusion. On day 7 of the infusion, 1490 genes were found to be differentially expressed (DE) compared to the day 0 control samples with FDR *p* < 0.05, vs. 105 genes on day 1 and 246 genes on Day 3. However, on day 8, after infusion was terminated for 24 h, only 428 genes were identified as differentially expressed compared to day 0 and only 107 genes continued to be identified by Day 14. Thus, the apparent differential expression of these genes is putatively a result of the single nutrient infused. Further, performing function and pathway analysis of the identified DE genes using IPA, we observe changes in digestive system development, and function pathways are among the primary functions of the DE genes, as well as immune response elements. Finally, primary transcription regulators such as PTH, JUN, WNT, and TNFRSF11B were identified as the activated upstream regulators for specific future focus. Using a serial biopsy approach we are able to identify differentially expressed genes from cow duodenal epithelial tissue in response to a short-term perturbation with infused starch hydrolysate.

## Introduction

Due to their central role in the absorption, processing, and assimilation of nutrients combined with relatively high metabolic activities, digestive tract tissues, and liver greatly influence maintenance energy requirements (1). Feed costs consistently represent >50% of production costs for dairy (2), thus incremental gains in feed efficiency through diet and ration management have a potentially large economic impact on producers. A great deal of research interest has been focused on these organs in ruminants. In fact, they have been shown to be affected by changes in metabolizable energy (ME) intake (1), protein intake (3, 4), nutrient restriction and/or realimentation (1, 6), and energy density of the diet (5, 6). Changes in mass associated with physiological state, when dietary energy intake is maintained, have been equivocal (7). Interestingly, it has been demonstrated (6) in growing steers that growth of the small intestine, liver, and forestomachs was the result of different processes following realimentation (hyperplastic growth, hypertrophic growth, and both, respectively). Small intestinal growth responses generally appear to be due to increases in cell number across a variety of dietary treatments, including nutrient restriction (4, 6).

The response of the rumen and small intestine to increased physiological demand for nutrients appears to be due largely to increased mass because of cell proliferation (4). Use of proliferation indices, including BrdU incorporation, Ki67 antigen staining, and tritiated thymidine incorporation assays, do not appear to be sufficiently sensitive to allow for accurate prediction of tissue proliferation status in the lactating dairy cow (8). Given that transit time through the cell cycle in the intestine is short (2–3 d); (9), a small change in transit time through the cell cycle can elicit a large net effect on total tissue proliferation. The specific molecular mechanisms regulating this increase in intestinal mass are not well studied in ruminants largely due to a lack of repeated access to these tissues. Using duodenally cannulated dairy cows we are now able to obtain serial biopsies from the duodenum using gastrointestinal endoscopy tools concomitantly with direct delivery of partially hydrolyzed starch to mimic increased post-ruminal delivery of starch from a ration. Using this approach we can procure samples to elucidate the ontogeny of the transcriptomic responses to increased starch.

The transcriptome is the essential and functional part of the genome (10). In this study, using transcriptomics and bioinformatics, we have compiled an information rich dataset and identified a large number of candidate genes for future experimental focus. Moreover, for the first time, we have identified specific transcriptomic regulators and identified pathways altered by direct infusion of a single nutrient (starch) using real-time transcriptomic profiles of the duodenal epithelium.

## Materials and Methods

All animal procedures were conducted under the approval of the Beltsville Location Institutional Animal Care and Use Committee (Protocol #15-008).

### Animals, Treatments, and Sampling

Six multiparous Holstein cows fitted with duodenal and ruminal cannulae were sampled during the dry period. The cows were fed standard diets *ad libitum* as a Total Mixed Ration (TMR; 50% corn silage and 50% concentrate at a dry matter basis) with free access to fresh water. Ruminal cannulas purchased (10.2 cm interior diameter; Bar Diamond, Inc., Parma, ID) and T-shaped duodenal cannulas (i.d., 2.5 cm; Tygon tubing R-3603; Norton Co., Akron, OH) were made by fusing with cyclohexanone. Duodenal cannulas were placed approximately 15-cm distal to the pylorus.

Prior to initiating infusions and sampling, cows were moved to a tie stall barn for adaptation and acclimation for at least 5 days prior to the infusion experiment. Infusion of a partially hydrolyzed starch solution (to achieve 20% MEI coming from infusate) was initiated immediately following 0 h sampling and thereafter continued for 168 h (7 days) at a rate of 5.0 L/d of a corn starch solution prepared as described by Bauer et al. (11) and stored (−20°C) until infused. After 168 h infusion, cows were maintained on the standard ration without starch infusion for an additional 168 h. Duodenal biopsies (20–30 mg/biopsy) were serially collected at 0, 24, 72, and 168 h of infusion, and 24 and 168 h post infusion through the duodenal cannula using sterile biopsy forceps aided by a Pentax EC-383IL colonoscope (PENTAX of America, New Jersey, 07645-1782 USA). Biopsies were rinsed in saline and placed into RNAlater and handled per manufacturer recommendations. Samples were stored frozen (−80°C) until sequencing.

### RNA Sequencing and Bioinformatic Analysis

RNA-sequencing: Samples were isolated using Qiagen RNeasy Plus Mini Kit (Qiagen). The quality check was performed using Tapestation RNA HS Assay (Agilent Technologies, CA, USA) and quantified by Qubit RNA HS assay (ThermoFisher). Ribosomal RNA depletion was performed with Ribo-zero Magnetic Gold Kit (Catalog number MRZG12324, Illumina Inc., San Diego, CA. Samples are randomly primed and fragmented based on manufacturer's recommendation (NEBNext® Ultra™ RNA Library Prep Kit for Illumina®). The first strand is synthesized with the Protoscript II Reverse Transcriptase with a more extended extension period (40 min for 42°C). All remaining steps for library construction were used according to the NEBNext® Ultra™ RNA Library Prep Kit for Illumina®. Illumina 8-nt dual-indices were used. Samples were pooled and sequenced on a HiSeq with a read length configuration of 150 PE.

Bioinformatic Analysis: Raw data quality assessment and preprocessing. During the library preparation and sequencing, artificial/technical biases, as well as sample contamination, could be introduced and affect the accuracy of the downstream statistical analysis (Mapping statistics are provided in Supplementary Table 1). We performed a thorough quality assessment using FASTQC (version v0.11.3). Sequence alignment: STAR (version 2.5.2b) (12), a splice aware aligner, was used to perform the RNA-Seq alignment. The UMD3.1 and UMD3.1.90 from Ensembl were used as genome reference and annotation reference, respectively during the alignment. Then dupRader and Picard CollectRnaSeqMetrics (version 2.10.5) were used to evaluate duplicates level and overall alignment performance.

Gene Expression Estimation and Differential Expression Analysis: We used HT-Seq (version 0.6.0) (13) to calculate the per gene expression count and DE-Seq (14) was used to estimate the differentially expressed genes. Some quality control assessments, as well as downstream exploratory analysis, were primarily performed using R package including but not limited to mixOmics, clusterProfiler, topGO, DOSE, pathview, and org.Bt.eg.db.

Functional Annotation of Differentially Expressed Genes: Ingenuity Pathways Analysis (IPA, Qiagen) was used to further identify the molecular processes, molecular functions, and genetic networks affected by starch infusion through analysis of the identified differentially expressed genes. As an integrated analysis software, IPA is a software application that enables users to identify the biological mechanisms, pathways, and functions most relevant to their experimental datasets or genes of interest. The “core analysis” function included in the IPA software was used to interpret the differentially expressed data, which included identification of probable biological processes, canonical pathways, upstream transcriptional regulators, and gene networks responding to the starch infusion. The temporally dynamic changes in gene activities during starch infusion were also compared using IPA.

## Results

### RNA-seq Revealed Dynamic Changes in Global Gene Expression of Cattle Intestinal Epithelium During Infusion

**Figure 1:**
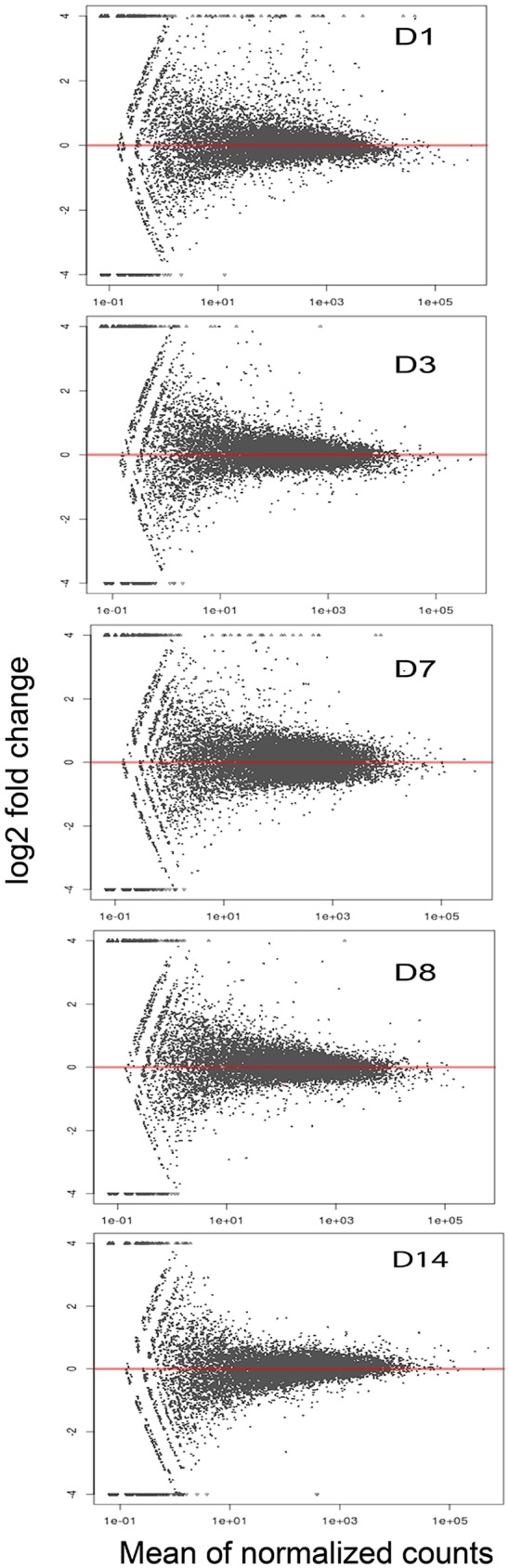
RNA-seq MA Plots for mean six normalized samples show differential expression of genes at five sampling time points.

**Figure 2:**
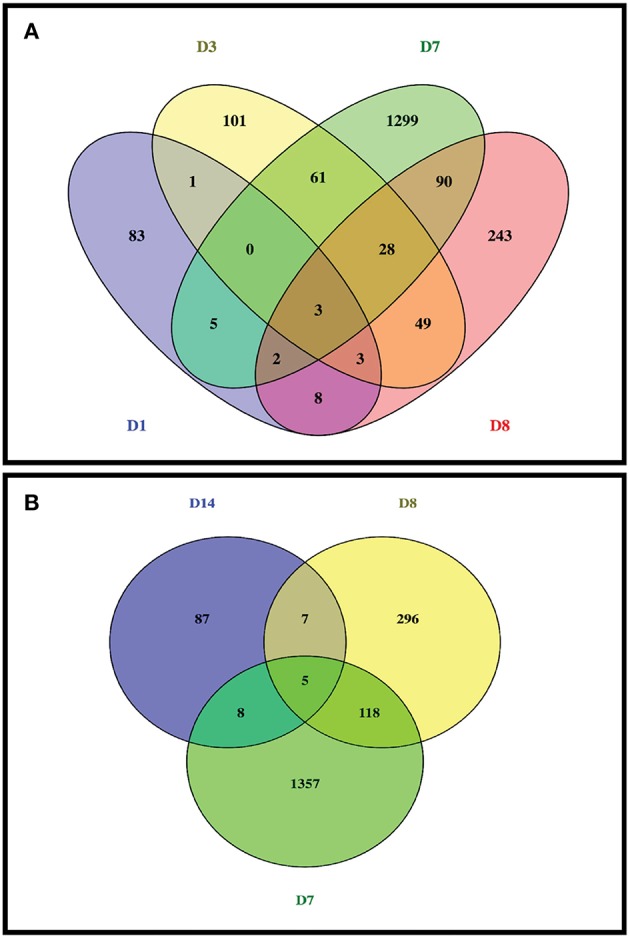
Venn diagram of diferentially expressed genes induced by partially hydrolysed starch infusion at different sampling points. **(A)** Sample points of D1, D3, D7, and D8; **(B)** Sample time points of D7, D8, and D14.

From RNA sequencing reads of 30 intestinal epithelial samples (6 animals with 5 sampling time points on Day 0 (D0), Day 1 (D1), Day 3 (D3), Day 7 (D7), and Day 14 (D14)), a total of varied from 15,643 to 16,845 genes were detected from at least one of the RNA sequenced samples, and highest number of genes (16,845) was detected in D7 of starch hydrolysate sampling. Likewise, total gene transcripts detected attained apeak on D7 (Table 1). In comparison to pre-infusion at 0 h, a total of 1,795 DE genes were identified at least once at the different sampling time points at a stringent cutoff of FDR <0.01 as impacted in the biopsies of intestinal epithelium in response to the starch infusion. The apparent maximal effect of starch hydrolysate infusion was observed on day 7 (Table 1; Figure 1) where 1,490 genes were found to be differentially expressed (DEGs) with FDR *p* < 0.05, compared to 105 genes on D1 and 246 genes on D3. After the partially hydrolyzed starch infusion was terminated, on D8 (one-day post infusion), only 428 genes were identified differentially expressed compared to day 0 and only107 genes on D14 (7-day post-infusion). While numerous genes are overlapping across different time points among these impacted genes, 78 genes were responsive only on D1, 148 genes were only on D3, and 1,380 genes were impacted only on D7. This occurred in such a way that most of the impacted genes are only represented at one or two sampling points. The overlapping and specific responding genes at the different sampling points were illustrated in a Venn diagram (Figure 2A).

**Table 1:** Profiling of transcriptome and responses of the intestinal epithelium to starch hydrolysate direct infusion^a^.

**Time point**	**Total gen transcripts detected**	**Number of degs^**b**^**	**Up-regulated gene**	**Down-regulated gene**
D1	15,643	105	44	61
D3	15,646	245	159	86
D7	16,845	1,490	851	639
D8	16,839	428	270	158
D14	16,708	107	25	82

a *In comparison to pre-infusion at 0 h, genes were identified to be impacted in the intestinal epithelium by partially hydrolysed starch infusion at a stringent cutoff of FDR <0.01*.

b *DEGs: differentially expressed genes*.

### Gene Ontology (GO) Enrichment Analysis of Differentially Expressed Genes Impacted by Starch Infusion

Gene ontology (GO) enrichment analysis of the differentially expressed genes was performed to further clarify the putative functions affected by starch infusion within each sampling day compared to control samples from Day 0. Changes of GO terms from enrichment analysis across sampling days support the concept of a coordinated and dynamic response temporal response to starch infusion by the duodenal epithelial transcriptome. The top GO terms in the biological processes significantly enriched in differentially expressed genes from each sampling point are listed in Table 2. While there are 105 DEGs for the D1 sampling, no enriched GO terms were identifiable. By day 7 of starch infusion the most significantly enriched GO terms included: biological processes, metabolic processes, cellular processes, primary metabolic processes, and organic substance metabolic processes. The top GO terms enriched for DEGs at each sampling point (D3, D7, D8, and D14) are presented in Table 2. All of the genes present for each GO terms in the dataset are listed in Supplementary Table 2. In addition to the biological processes, GO terms in molecular activities categories were also analyzed. Interestingly, most GO terms in molecular activity in the 36 samples were related to the following molecular functions; Cytokine receptor binding, catalytic activity; and molecular binding (Table 3; Supplementary Table 3), as we found for biological functions, on D1 there is no enriched GO term detected for molecular functions.

**Table 2:** Top GO terms in biological processes significantly impacted temporally by partially hydrolysed starch infusion^a^^,^^b^.

**GO ID**	**Description ion**	**Gene ratio**	**BgRatio**	***P*-value**	***P*-value adjusted**	***q*-value**
**D3**						
GO:0019221	Cytokine-mediated signaling pathway	8/58	76/5,527	9.7 × 10^−7^	0.0009	0.0008
GO:0071345	Cellular response to cytokine stimulus	9/58	106/5,527	1.21 × 10^−6^	0.0009	0.0008
GO:0034097	Response to cytokine	9/58	122/5,527	3.96 × 10^−6^	0.0020	0.0017
GO:0046627	Negative regulation of insulin receptor signaling pathway	3/58	7/5,527	3.73 × 10^−5^	0.0131	0.0107
GO:1901700	Response to oxygen-containing compound	11/58	257/5,527	5.76 × 10^−5^	0.0131	0.0107
**D7**						
GO:0008150	Biological process	421/421	4,837/5,527	3.72 × 10^−26^	< 0.0001	< 0.0001
GO:0008152	Metabolic process	317/421	3,464/5,527	7.17 × 10^−9^	< 0.0001	< 0.0001
GO:0009987	Cellular process	360/421	4,158/5,527	6.23 × 10^−8^	< 0.0001	< 0.0001
GO:0044238	Primary metabolic process	256/421	2,843/5,527	3.64 × 10^−5^	0.0263	0.0258
GO:0071704	Organic substance metabolic process	263/421	2,970/5,527	1.03 × 10^−4^	0.0428	0.0420
**D8**						
GO:0008150	Biological process	125/125	4,837/5,527	4.7 × 10^−8^	< 0.0001	< 0.0001
**D14**						
GO:0065007	Biological regulation	24/27	2,624/5,527	7.79 × 10^−6^	0.0059	0.0049
GO:0050789	Regulation of biological process	23/27	2,445/5,527	1.33 × 10^−5^	0.0059	0.0049
GO:0050794	Regulation of cellular process	21/27	2,257/5,527	1.01 × 10^−4^	0.0302	0.0248
GO:0050896	Response to stimulus	18/27	1,832/5,527	3.64 × 10^−4^	0.0499	0.0411
GO:0007094	Mitotic spindle assembly checkpoint	2/27	8/5,527	6.32 × 10^−4^	0.0499	0.0411

a *GO: gene ontology*.

b *All the time points (day) are compared against D 0 (baseline control); 2. Gene Ratio = the number of all genes assigned to this GO term to the number of significantly regulated genes that can be assigned to this GO term, BgRatio: ratio between the number of genes in the pathway and the total examined background of genes. P-value: for hypergeometric test; P-value adjusted: P-value for hypergeometric test adjusted for Benjamini-Hochberg correction*.

**Table 3:** Top enriched GO terms in molecular functions significantly impacted temporally by partially hydrolysed starch infusion^a^^,^^b^.

**GO ID**	**Description**	**GeneRatio**	**BgRatio**	***P*-value**	***P*-value adjusted**	***q*-value**
**D3**						
GO:0003674	Molecular function	54/54	4,363/5,527	2.65 × 10^−6^	0.0006	0.0005
GO:0005126	Cytokine receptor binding	6/54	83/5,527	1.38 × 10^−4^	0.0105	0.0094
GO:0042802	Identical protein binding	11/54	307/5,527	1.45 × 10^−4^	0.0105	0.0094
GO:0008009	Chemokine activity	3/54	17/5,527	5.44 × 10^−4^	0.0256	0.0229
GO:0005125	cytokine activity	5/54	75/5,527	7.58 × 10^−4^	0.0256	0.0230
**D7**						
GO:0003674	Molecular function	382/382	4,363/5,527	1.44 × 10^−41^	< 0.0001	< 0.0001
GO:0005488	Binding	304/382	3,268/5,527	1.33 × 10^−18^	< 0.0001	< 0.0001
GO:0003824	Catalytic activity	198/382	1,872/5,527	5.37 × 10^−14^	< 0.0001	< 0.0001
GO:0043167	Ion binding	179/382	1,678/5,527	1.58 × 10^−12^	< 0.0001	< 0.0001
GO:0046872	Metalion binding	119/382	1,066/5,527	5.99 × 10^−9^	< 0.0001	< 0.0001
**D8**						
GO:0003674	Molecular function	118/118	4,363/5,527	5.42 × 10^−13^	< 0.0001	< 0.0001
**D14**						
GO:0004872	Receptor activity	7/24	224/5,527	3.14 × 10^−5^	0.0050	0.0041
GO:0060089	Molecular transducer activity	7/24	293/5,527	1.74 × 10^−4^	0.0107	0.0089
GO:0008329	Signaling pattern recognition receptor activity	2/24	132/4,887	2.68 × 10^−4^	0.0107	0.0089
GO:0038187	Pattern recognition receptor activity	2/24	132/4,887	2.68 × 10^−4^	0.0107	0.0089
GO:0038023	Signaling receptor activity	5/24	188/5,527	1.08 × 10^−4^	0.0344	0.02879

a *GO: gene ontology*.

b *All the time points (day) are compared against D0 (baseline control); 2. GeneRatio = the number of all genes assigned to this GO term to the number of significantly regulated genes that can be assigned to this GO term, BgRatio: ratio between the number of genes in the pathway and the total examined background of genes. P-value: for hypergeometric test; P-value adjusted: P-value for hypergeometric test adjusted for Benjamini-Hochberg correction*.

### Functional Annotation of Differentially Expressed Genes Using IPA

**Figure 3:**
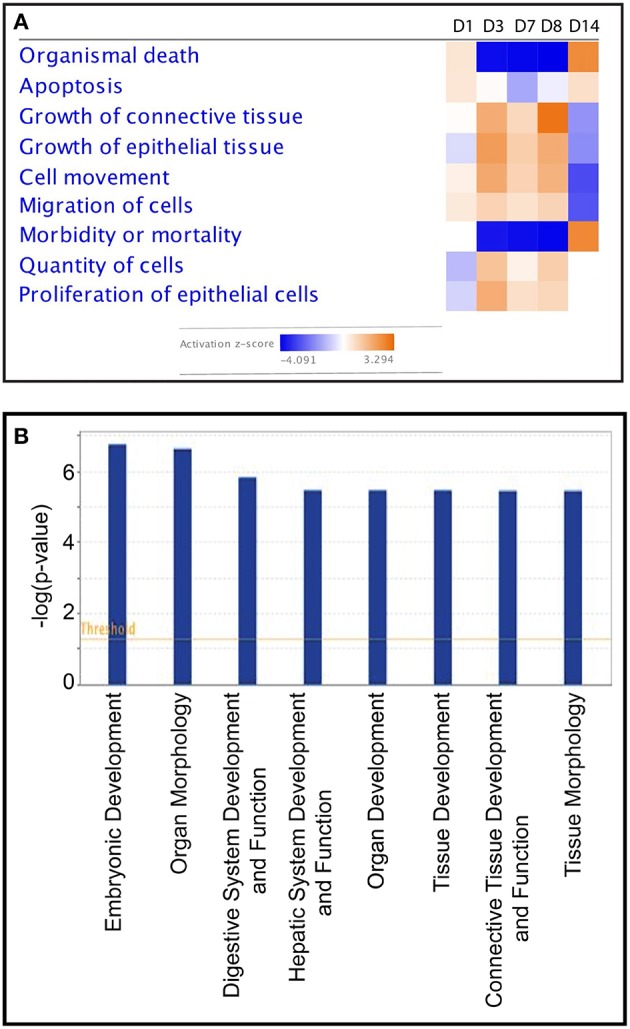
**(A)** Heatmap—comparison of the top biological functions impacted by partially hydrolysed starch infusion at the different samspling time points with activation z-score. **(B)** Top physiological functions of DEGs on D7.

**Figure 4:**
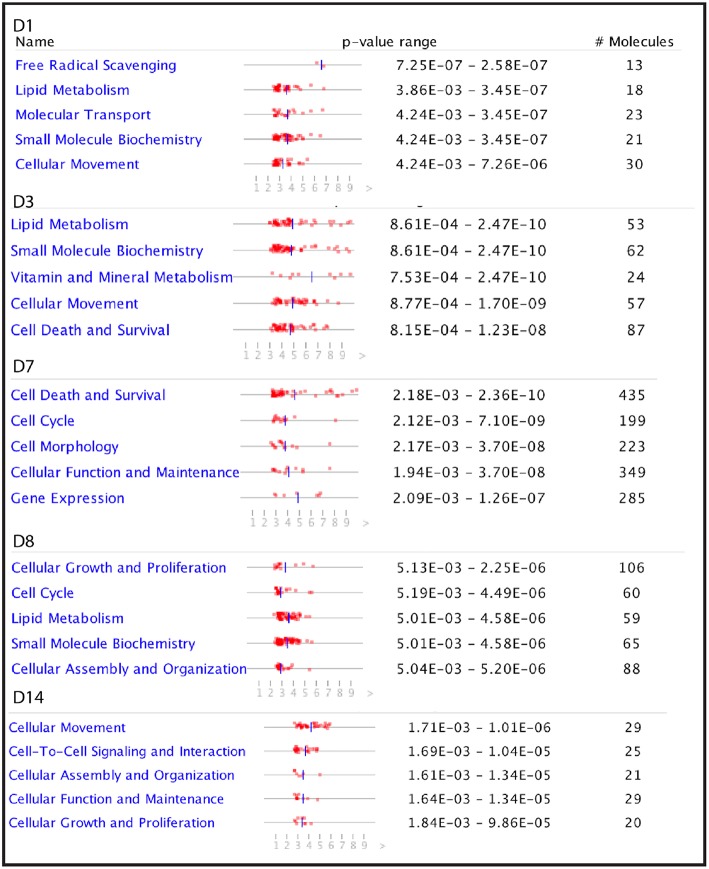
The top five molecular functions impacted by partially hydrolysed starch infusion at the different sampling points during the experiment.

To investigate further the biological functions affected by the starch infusion, Ingenuity Pathways Analysis (IPA) was utilized. Comparison analysis using IPA was performed to elucidate the dynamics and the tendency of the biological and molecular functions impacted by starch infusion through the experimental infusion period. The top affected functions of the identified DEGs at each sampling time point are presented in two figures, Figures 3, 4. In Figure 3A, the top biological functions impacted by starch infusion are presented in a heatmap according to their activation z-scores. The predominant positive biological functions impacted during the whole experimental course were the growth of connective tissue, the growth of epithelial tissue, and proliferation of epithelial cells. Consistently, the primary physiological functions of the DEGs on D7 are digestive system development and function related (Figure 3B). In Figure 4, top molecular functions significantly impacted by starch infusion are listed. The results were consistent with the GO enrichment analysis.

**Figure 5:**
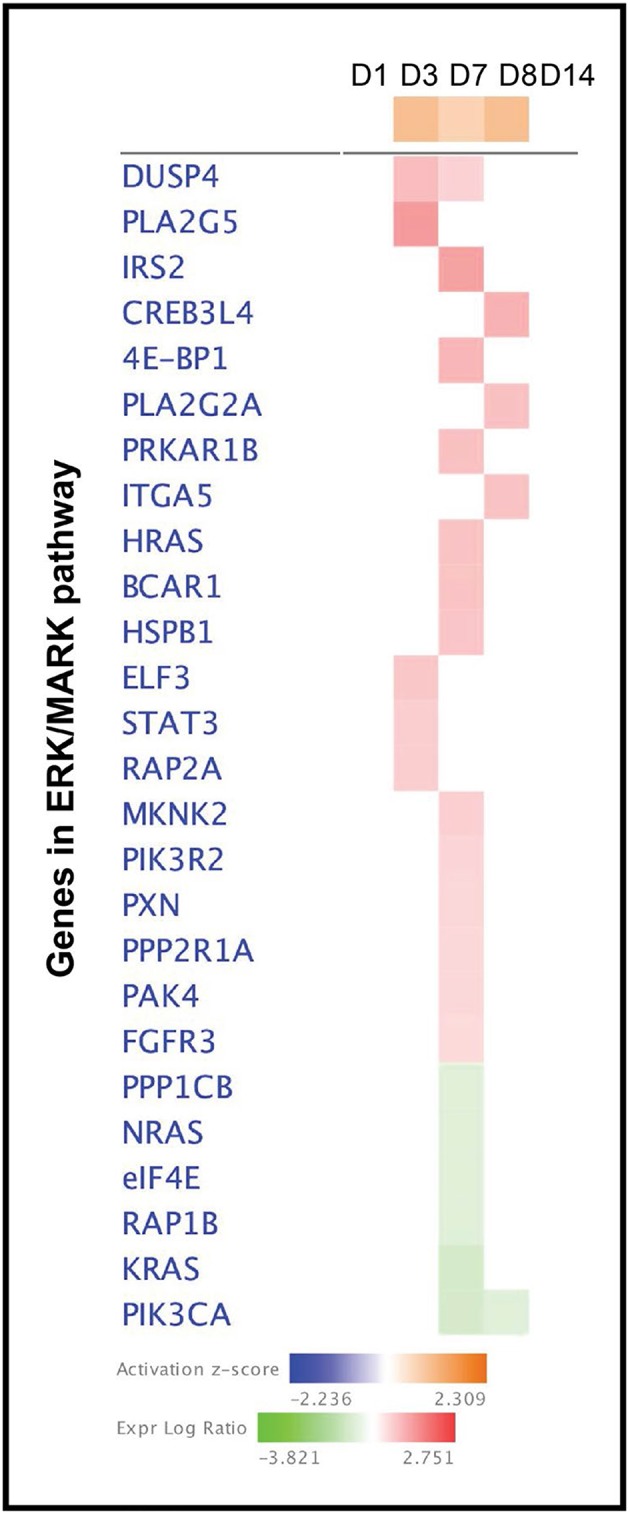
A heatmap shows the expression of the genes in ERK/MARK pathway induced by partially hydrolysed starch infusion.

In addition to the biological and molecular functions, IPA analysis revealed canonical pathways putatively affected as determined from the DEGs. Some essential canonical pathways were induced by starch-infusion such as ERK/MARK presented graphically in Figure 5. The heat map is used to visualize the pathway scores (Activation z-score) and the expression of the genes involved in the canonical pathways network. These heat maps can also show changes in relative expression across the five sampling time points (D1, D2, D7, D8, and D14) simultaneously. Figure 5 presents the activation z-score of ERK/MARK singling pathway with the expression of genes in ERK/MARK signaling pathway network. The activation z-score for this pathway is at its highest activation status on the D3, D7 which continues to D8 of starch infusion.

**Figure 6:**
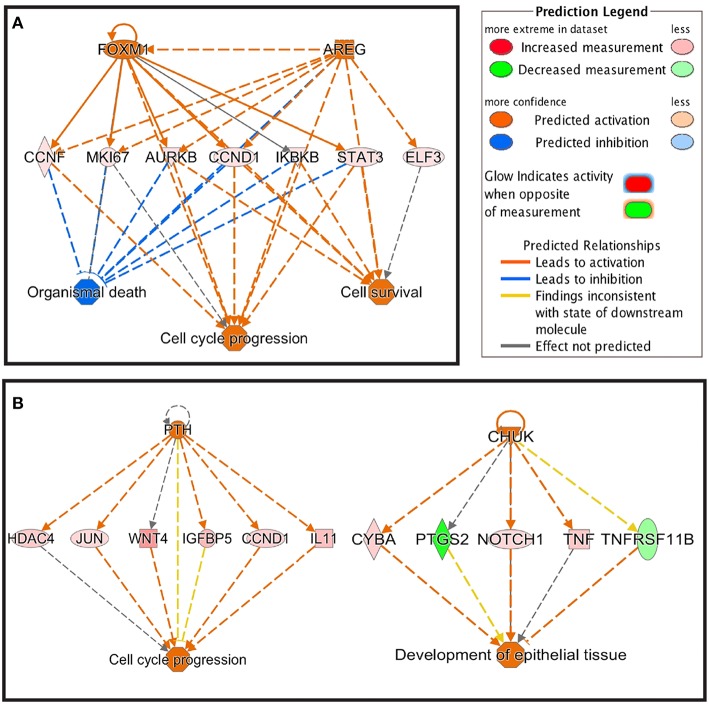
Regulator effects of FOXM1 and AREG on D3 **(A)** and PTH and CHUK on D7 **(B)**.

Using IPA analysis, potential upstream regulators of the DEGs in response to starch infusion were identified. Upstream regulators of the DEGs in this data set are identified based on known molecular actions from the literature and thus, may be involved with regulation of the response observed in the DEG identified. The top five upstream regulators at the different sampling time points are listed in Table 4. There is apparent overlap in the regulatory actions of these regulators with the affected cellular functions as illustrated in Figure 6. On D3, upregulated upstream regulators FOXM1 and AREG are overlapped with upregulated genes of CCNF, MKI67, AURKB, CCND1, IKBKB, STAT3, and ELF3 and the result would be effects on cell cycle progression and cell survival activities. Similarly, on D7, enhanced activities of upstream regulators PTH and CHUK would be expected to result in activation of cell cycle progression and development of epithelial tissue.

**Table 4:** Top upstream regulators and *P*-value of overlap predicted activation.

**Upstream regulator**	**Molecule type**	***P*-value of overlap**
**D1**		
TNF	Cytokine	3.90 × 10^−09^
Cg	Complex	2.71 × 10^−07^
IFNG	Cytokine	4.50 × 10^−06^
EGF	Growth factor	9.76 × 10^−06^
CDX1	transcription regulator	3.58 × 10^−05^
**D3**		
CFTR	Ion channel	2.46 × 10^−09^
PPARA	Ligand-dependent nuclear receptor	4.62 × 10^−09^
TLR4	Transmembrane receptor	1.39 × 10^−08^
MET	Kinase	2.99 × 10^−08^
TNF	Cytokine	9.90 × 10^−08^
**D7**		
MIR17HG	Other	1.39 × 10^−06^
INS	Other	3.26 × 10^−05^
THRB	Ligand-dependent nuclear receptor	8.61 × 10^−05^
EPAS1	Transcription regulator	1.76 × 10^−04^
IGFBP7	Transporter	2.17 × 10^−04^
**D8**		
ERBB2	Kinase	1.22 × 10^−11^
AREG	Growth factor	2.37 × 10^−07^
PPARA	Ligand-dependent nuclear receptor	4.56 × 10^−07^
TGFB1	Growth factor	6.73 × 10^−07^
EP400	Other	3.26 × 10^−06^
**D14**		
TNF	Cytokine	1.54 × 10^−08^
TGFB1	Growth factor	1.85 × 10^−06^
STAT3	Transcription regulator	5.43 × 10^−06^
GLI2	Transcription regulator	6.17 × 10^−06^
TP73	Transcription regulator	8.09 × 10^−06^

**Figure 7:**
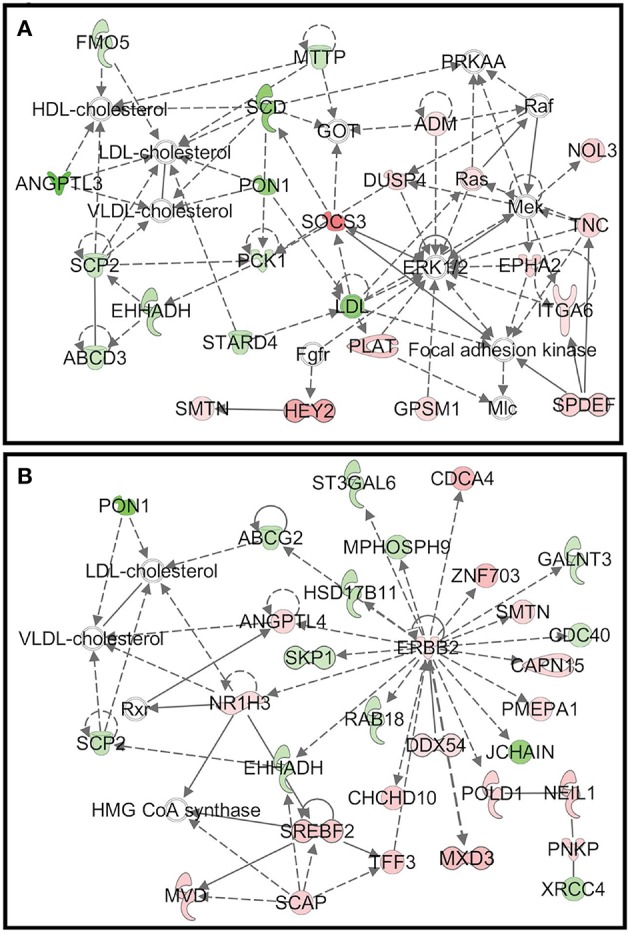
**(A)** The top biologically relevant network on D3 is associated with lipid metabolism, molecular transport, small molecule biochemistry and **(B)** the top biologically relevant network on D7 is associated with the biological functions of carbohydrate metabolism, lipid metabolism, and molecular transport.

As mentioned above, during the starch infusion, the most DEGs altered in duodenal epithelium were observed in the D7 samples. Functional network analysis identifies the biologically relevant networks based on the DEGs in response to starch infusion. The top biologically relevant network on D3 is associated with lipid metabolism, molecular transport, small molecule biochemistry (Figure 7A) and the top biologically relevant network on D7 is associated with the biological functions of carbohydrate metabolism, lipid metabolism, and molecular transport (Figure 7B).

## Discussion

The duodenal epithelium is a highly metabolically active tissue due to the functions it performs (absorption, transport and protection). In fact, total gastrointestinal tissues use a disproportionate amount of the energy used by the animal (about 25% of total oxygen consumption) given its relative size (about 6% of body weight). Additionally understanding the extent to which individual nutrients are used by gut tissues is important to assess net nutrient needs of the animal (15). Ruminal and abomasal starch hydrolysate infusions have been used previously to study the metabolism (11) and gene expression of intestinal epithelia (16–18). However, those studies were limited by the availability of technology at the time, and only a few genes were examined. In this report, coupling next generation sequencing and transcriptomic profiling approaches with a serial biopsy sampling scheme were used to investigate the changes in the golobal transcriptome during an adaptation of intestinal epithelia of dairy cattle to a single nutrient, starch hydrolysate, by direct duodenal infusion.

The transcriptome is known to have distinct profiles unique to cell type, developmental stages, and health status (10). RNA-sequencing (RNA-seq) has been widely used as a highly reliable tool for unbiased analysis of transcriptome changes within cells and tissues (19). Using a direct biopsy technique aided by a Pentax EC-383IL colonoscope, we were also able to serially collect the duodenal epithelial samples throughout a single nutrient infusion experimental protocol lasting 14 day. Next we assembled the transcriptome and compared gene expression patterns and thus, can assess if temporal impacts on the duodenal epithelial transcriptome induced by starch hydrolysate infusion in dairy cattle are detectable.

Notably, the transcriptomic response occurred in a pattern where a majority of the DEG only represented at one or two sampling points used, potentially indicating a coordinated temporal pattern of changes by intestinal epithelial transcriptome induced by starch hydrolysate. After 7 d of the infusion, 1,490 genes were identified as differentially expressed (with FDR *p* < 0.05), compared to only 105 genes on D1 and 246 genes on D3. Moreover, after terminating the infusion for a day, (D8; one day post infusion), a maked decrease in DEG (only 428 DEGs) was observed compared to D0 and by D14 (7 d post termination fo infusion) only 107 genes were different from D0. Thus, it appears that differential expression of genes during infusion is putatively the result of the starch hydrolysate infusion. Mechanistically we are not able to completely rule out that other physical or environmental factors changed over time, however, the experimental design minimized other factors by maintaining cows on a consistent TMR throughout the experiment.

Regulation of gene expression within the intestinal epithelium, as with other tissues, is complex and controlled by various signaling pathways that regulate the balance between proliferation and differentiation (9). We have previously identified in sheep nutrient use efficiency and body composition experiments that when nutrient density is increased (increased concentrate) by altering forage and concentration ratio in the ration, there is an increase in intestinal epithelial cell mass (5). Indeed, the DEG observed in the current experiment are predictive of an increased cell proliferation in response to the starch. Positively affected biological functions identified as impacted during the infusions were growth of connective tissue, the growth of epithelial tissue, and proliferation of epithelial cells (Figure 3). Clearly, these biological functions are consistent with the major molecular functions induced by starch infusion, and given the nature of the treatment, appear to be treatment specific.

Consistently, the genes in the ERK (extracellular-regulated kinase)/MAPK (mitogen activated protein kinase) signaling pathways are activated by the infusion protocol used. The ERK/MAPK pathway is a crucial pathway that transduces cellular information on meiosis/mitosis, growth, and differentiation within a cell. The mitogen-activated protein kinases (MAPK) signaling pathway is shared by four distinct cascades, including the extracellular signal-related kinases (ERK1/2), Jun amino-terminal kinases (JNK1/2/3), p38-MAPK, and ERK5 (20). ERK is also translocated into the nucleus where it induces gene transcription by interacting with transcriptional regulators like ELK-1, STAT-1 and−3, ETS, and MYC. ERK activation of p90RSK in the cytoplasm leads to its nuclear translocation where it indirectly induces gene transcription through interaction with transcriptional regulators, CREB, c-Fos, and SRF (21). This all consistent with the potential for ERK/MAPK pathway to have an important role in the epithelial response to increased luminal starch.

Our data also identified a number of immune system markers such as TNF and cytokines, which are differentially expressed after infusion of a partially hydrolyzed starch solution. This could be an indication that gut immune cells are impacted by the influx of the starch directly or may play an important role in absorption, metabolism, and transport of glucose by the epithelial tissue. The GO term analysis likewise indicates the appearance of an immune response as cytokine-mediated signaling pathway is significantly perturbed by the treatment. The gastrointestinal epithelium has a large number of immune cells integrated within the tissue presumably to enhance defense against disease-causing microbes. Recent reports have demonstrated the presence of specific types of immune cells distributed throughout the intestinal epithelium as intraepithelial lymphocytes (22, 23). They further demonstrated that in addition to their immune functions, the cells have an integral role in the control of metabolism through regulation of hormones released in response to feed consumption. This interesting finding supports the contention that immune cells may be involved in the control of metabolism. Further evidence of a metabolic role of these cells is not only their relative abundance in the sections of the intestine where nutrient absorption occurs, but also that they express genes associated with metabolism even in the absence of infection (22).

By performing function and pathway analysis of DE genes using IPA, we found, perhaps unsurprisingly, that digestive system development and function are among the primary functions of the DEGs identified. Furthermore, primary transcription regulators such as PTH, JUN, WNT, and TNFRSF11B were identified as the activated upstream regulators (Figure 6). Previous research provided indications that WNT signaling is important for proliferation of the intestinal epithelium (24). The enhanced activities of upstream regulators PTH and CHUK would be expected to result in activation of cell cycle progression and development of epithelial tissue. These results at the transcription level of integration demonstrate the responsive nature of the intestinal epithelial tissue to a single nutrient delivered on the luminal side with no other changes in diet. As outlined earlier, changes in gastrointestinal, and specifically epithelial, mass are known to be a response to alteration in diet and ration delivery in productive ruminants. The transciptiomic changes of these biopsied tissue are likely the necessary response to maintain epithelial homeostasis in the face of a changing nutrient supply.

Unsurprisingly given starch hydrolysate was the nutrient infused, the top functional network identified is specifically related to functions such as carbohydrate metabolism, lipid metabolism, and molecular transport (Figure 7). Changes in these functions as the top network reflect the temporal transcriptomic response of duodenal epithelium to the starch hydrolysate infusion. The networks also explored functional interactions among DEGs.

In summary, transcriptomic profiling with next-generation sequencing and bioinformatics were utilized to accelerate our understanding of the multiple levels of regulation ongoing in duodenal epithelial transcriptome induced by starch infusion. Use of direct infusion of a single nutrient, in combination with serial biopsy technique, has facilitated real-time sample collection and thus the ability to assess the temporal impacts on the duodenal epithelial transcriptome induced by starch hydrolysate infusion. Moreover, direct duodenal infusion of starch hydrolysate induces measurable transcriptomic responses in epithelial tissue of cattle intestine in short-term experiments that will ultimately facilitate a better understanding of the regulation of this tissue level response. Several important pathways and regulator mechaisms have been identified for future experimental focus. The use of transcriptomic profiling provides comprehensive gene expression information for improving our understanding of the molecular mechanisms involved in the intestinal functions, as well as maintaining epithelial homeostasis of cattle intestine.

## Ethics Statement

This study was carried out in accordance with the recommendations of the Institutional Animal Care and Use Committee for the Beltsville Location. The protocol was approved by the Beltsville Location Institutional Animal Care and Use Committee.

## Author Contributions

C-JL oversaw sample analysis, bioinformatics, prepared figures and tables, and prepared manuscript with RB. SL participated in statistical analysis and bioinformatics. MR-G participated in experiment, sample collection, analysis of samples, and manuscript review. RB developed experimental design, conducted all aspects of experiment, sampling, analysis, interpreting of results, and writing manuscript.

### Conflict of Interest Statement

The authors declare that the research was conducted in the absence of any commercial or financial relationships that could be construed as a potential conflict of interest.

## Supplementary Material

Supplementary Table 1 Mapping statistics of the RNA-Seq data used for profiling the transcriptome and response of the intestinal epithelium to starch hydrolysate direct infusion.

Supplementary Table 2 GO terms (Biological Process) enriched for DEGs.

Supplementary Table 3 GO terms (Molecular Function) enriched for DEGs.
