# Supplementary material for: Transcriptomic Profiling of Duodenal Epithelium Reveals Temporally Dynamic Impacts of Direct Duodenal Starch-Infusion During Dry Period of Dairy Cattle
Source: Front Vet Sci. 2019 Jul 2;6:214. doi: 10.3389/fvets.2019.00214 (PMC6614288; doi:10.3389/fvets.2019.00214)
Supplement: Supplementary Table 1 — Mapping statistics of the RNA-Seq data used for profiling the transcriptome and response of the intestinal epithelium to starch hydrolysate direct infusion. [file Table_1.DOCX]

| **A: Read count statistics*** | |  |  |  |
| --- | --- | --- | --- | --- |
| **Sample name** | **Read count** | **Paired, mapped pairs %** | **Paired, broken pairs %** | **Paired, not mapped %** |
| D0 RNA-Seq report | 63180384 | 75.83 | 8.61 | 15.56 |
| D1 RNA-Seq report | 50882544 | 84.24 | 11.1 | 4.66 |
| D3 RNA-Seq report | 58564124 | 84.43 | 9.98 | 5.59 |
| D7 RNA-Seq report | 67628036 | 81.14 | 9.88 | 8.98 |
| D8 RNA-Seq report | 40640174 | 80.25 | 10.98 | 8.77 |
| D14 RNA-Seq report | 87640548 | 75.56 | 19.41 | 5.02 |
|  |  |  |  |  |
| *For paired data, there are two reads in a pair. | |  |  |  |
|  |  |  |  |  |
| **B: Fragment counting statistics*** | |  |  |  |
| **Sample name** | **Mapped to genes %** | **Mapped to intergenic %** |  |  |
| D0 RNA-Seq report | 46.09 | 53.91 |  |  |
| D1 RNA-Seq report | 53.11 | 46.89 |  |  |
| D3 RNA-Seq report | 78.85 | 21.15 |  |  |
| D7 RNA-Seq report | 68.83 | 31.17 |  |  |
| D8 RNA-Seq report | 70.87 | 29.13 |  |  |
| D14 RNA-Seq report | 81.49 | 18.51 |  |  |
|  |  |  |  |  |
| *'Include broken pairs' counting scheme: A pair is counted as two, a single read as one. | | | | |
|  |  |  |  |  |
| **C: Strand specificity** | |  |  |  |
| **Sample name** | **Strand specific setting** | **Forward % of reads mapped** | **Reverse % of reads mapped** | **Ignored reads % (wrong strand)** |
| D0 RNA-Seq report | Both | 50.22 | 49.78 | 0 |
| D1 RNA-Seq report | Both | 50.16 | 49.84 | 0 |
| D3 RNA-Seq report | Both | 50.32 | 49.68 | 0 |
| D7 RNA-Seq report | Both | 50.28 | 49.72 | 0 |
| D8 RNA-Seq report | Both | 49.97 | 50.03 | 0 |
| D14 RNA-Seq report | Both | 50.17 | 49.83 | 0 |
